# No difference in patient-reported satisfaction after 12 months between customised individually made and off-the-shelf total knee arthroplasty

**DOI:** 10.1007/s00167-022-06900-z

**Published:** 2022-02-12

**Authors:** Séverin Wendelspiess, Raphael Kaelin, Nicole Vogel, Thomas Rychen, Markus P. Arnold

**Affiliations:** 1 Practice LEONARDO, Hirslanden Clinic Birshof, Reinacherstrasse 28, 4142 Münchenstein, Switzerland; 2 Faculty of Medicine, University of Basel, Basel, Switzerland

**Keywords:** Arthroplasty, Replacement, Knee, Custom, Patient-specific, Patient-reported outcome measure, Patient satisfaction

## Abstract

**Purpose:**

A subset of patients is usually not satisfied after a total knee arthroplasty (TKA). Customised individually made (CIM) TKA are deemed to overcome drawbacks of classical off-the-shelf (OTS) TKA, but evidence is still sparse. The aim of this study was to compare satisfaction of patients with CIM and OTS TKA.

**Methods:**

This prospective cohort study compared clinical and patient-reported outcome measures (PROM) between patients with CIM and OTS TKA. The primary outcome was patient satisfaction after 12 months. Secondary outcomes were the Knee Society Score (KSS), the Knee injury and Osteoarthritis Outcome Score (KOOS), the Forgotten Joint Score (FJS-12) and the EQ-5D-3L after 4 and 12 months.

**Results:**

Data were analysed from 74 CIM TKA and 169 OTS TKA between January 2017 and September 2020. Patients with CIM TKA were slightly younger, more often male, had a lower body mass index, a lower KSS and partially higher preoperative PROMs.

Patient satisfaction after 12 months was high and comparable (CIM 87%, OTS 89%). All PROMs improved for both groups (*p* < 0.001) and did not differ after 12 months (*p* > 0.063). The majority of patients improved above the minimal important difference (range 65 to 89%) and reported a clear overall improvement (CIM 86%, OTS 87%). The postoperative KSS, notably regarding knee stability, was higher for CIM TKA (*p* < 0.001).

**Conclusion:**

No difference was found in patient satisfaction between CIM and OTS TKA after 12 months. In both groups, patient satisfaction was high and PROMs improved considerably.

**Level of evidence:**

II, prospective cohort study.

**Supplementary Information:**

The online version contains supplementary material available at 10.1007/s00167-022-06900-z.

## Introduction

About 20% of patients are not satisfied after a total knee arthroplasty (TKA) [6, 12, 15]. A variety of predictors for dissatisfaction have been identified: female sex, lower grade of osteoarthritis, implant-related reasons, mental health problems, unfulfilled expectations, postoperative pain and limited function are only some of these [12, 15, 21, 29]. Moreover, the number of TKA is rising among younger and more active patients with high functional expectations [31].

Although TKA is a common, safe and cost-effective treatment for end-stage knee osteoarthritis [3, 12], the procedure does not entirely restore normal biomechanics and functional limitations may occur [26]. Classical off-the-shelf (OTS) TKA can cause implant overhang, malalignment and abnormal kinematics [20, 41]. One reason may be the high variability of knee phenotypes between individuals, ethnicities and sex [4, 7, 13, 19, 22]. Customised individually made (CIM) TKA have been developed to overcome these problems and improve outcome after TKA [5, 17, 44]. CIM TKA incorporate a bone-preserving approach using custom planning images, implants and instrumentation [44]. The personalised approach respects the patient’s individual knee anatomy and eliminates implant sizing compromises [18, 45]. Disadvantages of CIM TKA are a prolonged waiting time for the manufacturing, a higher radiation exposure [44] and limited intraoperative implant options [45].

To get a better understanding of the patients’ perspective, their satisfaction after TKA and potential problems in daily life, an evaluation of patient-reported outcome measures (PROM) is necessary. CIM TKA are relatively new, available in the US and Europe since 2011 [9] and at our hospital since 2015. First results are promising in terms of bone preservation [45], ligament balancing [45], alignment [2, 54] and patient satisfaction [32, 37]. However, sufficient data are still lacking: comparative studies are sparse and randomised controlled trials on this topic are absent [25, 51]. A prospective study comparing PROMs of patients with CIM and OTS TKA has not yet been published.

The aim of this study was to analyse patient-reported satisfaction; further PROMs and clinical outcome of patients with CIM TKA were compared to OTS TKA. Our hypothesis was that CIM TKA are superior to OTS TKA.

## Materials and methods

### Study design, setting and recruitment

This is a single-side, observational, prospective cohort study. The study is in accordance with the World Medical Association Declaration of Helsinki [48].

All patients were recruited in our medical practice, based in a private hospital, and gave their written informed consent. Since 2017, all patients scheduled for any type of knee arthroplasty were asked to complete a set of PROMs. Patients with insufficient knowledge of German, English, French or Italian were excluded. Details regarding recruitment and procedures are published in the study protocol [52]. The current study included consecutive patients with a primary cruciate-retaining CIM TKA (iTotal CR G2, ConforMIS Inc., Bedford, MA, US) or cruciate-retaining OTS TKA (Attune^®^ CR mobile-bearing, DePuy Synthes, Raynham, MA, US) who completed PROMs before the surgery and after 12 months. Patients with revision surgery or major re-operation on the affected knee were excluded. All TKA were performed between January 2017 and September 2020 by MPA (CIM and OTS) and by RK and TR (OTS), all of them well-experienced senior surgeons. CIM TKA patients chose their surgeon (MPA) accordingly because of their interest in the new technology. In rare cases, the patient was made aware of the possibility of a CIM TKA because of a marked joint line obliquity (tibial mechanical angle of ≤ 84° on long-leg radiographs) with an obvious anatomical difference in shape between the medial and lateral femoral condyles or hypoplasia of the lateral femoral condyle [13].

### Surgical technique

All patients had the same peri- and postoperative anaesthesia and pain management protocol. Preoperative, all patients received 1 g of tranexamic acid and a single-shot antibiosis with a cephalosporin intravenous. All surgeries used a medial parapatellar approach without tourniquet. Soft tissue release was only performed to an extent that was needed for an appropriate ligament balancing. For both, CIM and OTS TKA, spacer blocks are applied to adjust the soft tissue balancing process. A lateral (in valgus knees) or medial release (in varus knees) was used accordingly to achieve the ideal ligament balancing status. No more than grade one release was needed. Patellar resurfacing was only done in cases with severe patellofemoral arthritis with lateralised patella (i.e. bone on bone, especially if the patellofemoral joint surface was uneven). Both implants apply mechanical alignment.

The CIM implants are based on a preoperative computed tomography. Subsequently, the implant is manufactured and the surgeon provided with individualised instruments and a planning overview (iView^®^). The detailed surgical technique is described elsewhere [45]. The planning algorithm of the CIM TKA results in a hip–knee–ankle angle of 180° and a limited joint line obliquity provided by uneven medial and lateral inlay heights. For OTS TKA, a natural slope and rotation along the grinding marks on the arthritic tibial plateau is sought, followed by resection of the tibial plateau. After determining femoral rotation by the intramedullary balancer, the distal femur is resected first (extension gap). Subsequently, the posterior (flexion gap) and anterior femur condylar cut is made.

All patients had the same postoperative rehabilitation protocol with immediate full weight-bearing on crutches until sufficient muscular stabilisation and a proper gait pattern was achieved.

### Follow-up and outcome measures

Data were collected before the surgery and during routine control visits after 4 months and 12 months using Research Electronic Data Capture (REDCap^®^). Patients’ characteristics were extracted from the medical records. The surgeon graded the degree of osteoarthritis according to the Kellgren and Lawrence classification from 0 (no osteoarthritis) to 4 (severe osteoarthritis) [16] and completed the objective part of the Knee Society Score (KSS) ranging from 0 (worst) to 100 (best) points [40]. Comorbidities were classified according to the American Society of Anesthesiologists (ASA) from ASA I (normal healthy) to ASA V (moribund) [1].

The primary outcome was patient satisfaction after 12 months assessed on a five-point Likert scale (very satisfied, satisfied, neutral, unsatisfied or very unsatisfied). Secondary outcomes were all other PROMs: the Knee injury and Osteoarthritis Outcome Score (KOOS), the Forgotten Joint Score (FJS-12), the EQ-5D-3L, overall improvement and willingness to undergo the surgery again.

The KOOS comprises five subscales on pain, symptoms, activities of daily living, sports and quality of life on scales from 0 (worst) to 100 (best) points [34]. The FJS-12 captures the patient’s ability to forget the artificial joint in everyday life on a scale from 0 (worst) to 100 (best) points [50]. The EQ-5D-3L measures health-related quality of life ranging from 0 (worst) to 1 (best) and includes a visual analogue scale (VAS) from 0 (worst imaginable health) to 100 (best imaginable health) [11]. Overall improvement was measured on a seven-point Likert scale (very much better, substantially better, a little better, no change, a little worse, substantially worse or very much worse) and patients were asked if they would undergo the surgery again (yes or no).

Postoperative complications such as thromboembolic events, infections, re-operations, revisions or death were recorded as adverse events. Revision was defined as a second surgery to replace some or all parts of the original TKA.

### Ethical approval

The study was approved by the local ethics committee (reference: 2016-01777) [46].

### Statistics

The statistical evaluation was done with IBM SPSS statistics for Windows, Version 28, Armonk, NY: IBM Corp and R, Version 4.0.5 [30]. Descriptive statistics are presented with means and standard deviation (SD) for continuous variables, frequency counts and percentages for categorical variables. Differences between pre- and postoperative data were tested with paired t-test for continuous variables and Wilcoxon signed rank test for categorical variables. To improve the interpretability of PROM results, the proportion of patients whose PROMs improved more than the minimal important difference (MID) was calculated. The MID is defined as the smallest difference in a score that patients perceive as important [38]. The following MID cutoff values were applied: 10.7 for KOOS symptoms, 16.7 for KOOS pain, 18.4 for KOOS activities of daily living, 12.5 for KOOS sports, 15.6 for KOOS quality of life [23], 10.8 for the FJS-12 [10, 14] and 0.15 for EQ-5D-3L [10].

Differences in subgroups were measured with unpaired t-test for continuous variables and with Mann–Whitney *U* test or Chi-Square test for categorical variables. Bivariate linear correlations were analysed with the Pearson test for continuous variables and the Spearman test for categorical variables. The correlation effect sizes were classified as low (*r* = 0.1), medium (*r* = 0.3) or strong (*r* = 0.5) [8].

The a priori power calculation is based on the KSS after 4 months. When the study was planned, reliable data on patient satisfaction or any other PROM after 12 months were not available. The calculated effect size was 0.5 and resulted in a sample size of 64 patients per group to assure a power of 0.8 with two-sided alpha = 0.05.

## Results

**Fig. 1:**
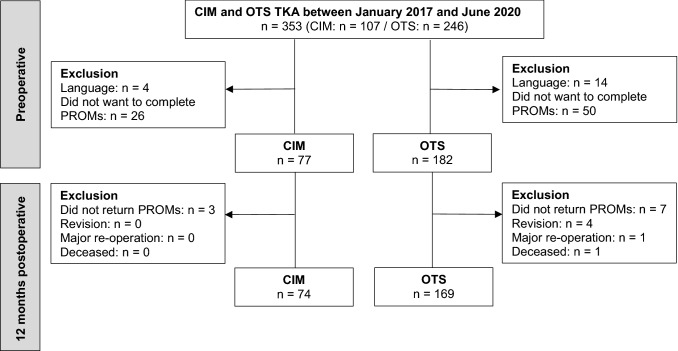
Flow chart of recruitment. *CIM* customised individually made, *OTS* off-the-shelf, *TKA* total knee arthroplasty, *PROM* patient-reported outcome measure, *n* number

Overall, 77 CIM and 182 OTS TKA were recruited and data of 74 CIM (63 patients, 30 female) and 169 OTS TKA (155 patients, 93 female) was analysed. Details regarding recruitment are shown in Fig. 1. Response rate to complete PROMs was 79% and loss to follow-up after 12 months was 4%. Patients’ characteristics are described in Table 1. Participants and patients who did not want to complete PROMs did not differ regarding patients’ characteristics (*p* > 0.159). Patients with CIM TKA were younger, more frequent male, had a lower body mass index, a lower KSS and partly higher PROMs preoperative (Tables 1 and 2).

**Table 1:** Patients’ characteristics

	CIM *n* = 74	OTS *n* = 169	*p* value
Age, mean years (± SD)	67.1 (± 8.4)	69.7 (± 8.9)	0.019
Age < 65 years, *n* (%)	26 (35%)	51 (30%)	0.457
Body mass index, mean kg/m^2^ (± SD)	26.6 (± 3.4)	28.7 (± 4.9)	< 0.001
Sex, *n* (%)			0.035
Male	41 (55%)	68 (40%)	
Female	33 (45%)	101 (60%)	
Insurance, *n* (%)			< 0.001
Basic insurance	3 (4%)	101 (60%)	
Supplementary insurance	71 (96%)	68 (40%)	
Side, *n* (%)			0.579
Left	32 (43%)	80 (47%)	
Right	42 (57%)	89 (53%)	
Surgery, *n* (%)			0.110
Unilateral	52 (70%)	141 (83%)	
Bilateral	22 (30%)	28 (17%)	
Kellgren and Lawrence classification, *n* (%)			0.849
1	0 (0%)	1 (1%)	
2	1 (1%)	5 (3%)	
3	20 (27%)	43 (25%)	
4	53 (72%)	120 (71%)	
ASA classification, *n* (%)			0.153
I/II	63 (85%)	129 (76%)	
III	10 (14%)	39 (23%)	
IV/V	1 (1%)	1 (1%)	
Operation time, mean min (± SD)	84 (± 14)	84 (± 18)	0.823
Length of stay, mean days (± SD)	6.4 (± 1.1)	6.5 (± 1.1)	0.500

*CIM* customised individually made, *OTS* off-the-shelf, *n* number, *SD* standard deviation, *ASA* American Society of Anesthesiologists

**Table 2:** KSS and PROMs before and after the surgery

	Before surgery			4 months			12 months		
	CIM *n* = 74	OTS *n* = 169	*p* value	CIM *n* = 74	OTS *n* = 169	*p* value	CIM *n* = 74	OTS *n* = 169	*p* value
	Mean (± SD)		(95% CI)	Mean (± SD)		(95% CI)	Mean (± SD)		(95% CI)
KSS	52.8 (± 11.9)	57.1 (± 13.4)	0.018 (− 7.9 to − 0.8)	90.4 (± 7.7)	85.3 (± 9.1)	< 0.001 (2.7 to 7.6)	94.2 (± 7.5)	88.9 (± 8.6)	< 0.001 (2.8 to 7.7)
KOOS symptoms	51.2 (± 16.8)	46.0 (± 18.0)	0.018 (0.4 to 10.1)	68.6 (± 15.3)	69.9 (± 16.4)	0.543 (− 5.8 to 3.1)	77.8 (± 16.0)	79.2 (± 14.9)	0.507 (− 5.6 to 2.8)
KOOS pain	49.6 (± 16.4)	41.9 (± 13.7)	< 0.001 (3.7 to 11.7)	71.7 (± 16.9)	71.7 (± 16.8)	1.000 (− 4.7 to 4.7)	84.5 (± 16.1)	82.2 (± 15.8)	0.321 (− 2.2 to 6.6)
KOOS daily living	57.4 (± 15.8)	48.2 (± 15.7)	< 0.001 (4.8 to 13.5)	78.0 (± 14.8)	77.1 (± 14.0)	0.671 (− 3.1 to 4.8)	87.2 (± 13.8)	84.3 (± 14.5)	0.151 (− 3.1 to 6.8)
KOOS sports	23.4 (± 18.8)	19.9 (± 18.3)	0.198 (− 1.8 to 8.7)	49.9 (± 24.5)	51.1 (± 25.7)	0.764 (−8.9 to 6.6)	68.1 (± 21.8)	62.4 (± 27.3)	0.141 (− 1.9 to 13.1)
KOOS quality of life	25.1 (± 13.0)	24.6 (± 14.1)	0.399 (− 3.3 to 4.3)	56.8 (± 20.6)	58.4 (± 20.3)	0.577 (− 7.2 to 4.1)	71.9 (± 21.5)	69.8 (± 21.8)	0.495 (− 3.9 to 8.1)
FJS-12	17.6 (± 12.4)	13.5 (± 13.1)	0.026 (0.5 to 7.6)	48.2 (± 25.6)	41.7 (± 26.0)	0.078 (− 0.7 to 13.8)	67.3 (± 25.0)	60.0 (± 29.2)	0.063 (− 0.4 to 15.1)
EQ-5D-3L	0.615 (± 0.184)	0.614 (± 0.182)	0.983 (− 0.050 to 0.051)	0.826 (± 0.158)	0.780 (± 0.149)	0.066 (− 0.003 to 0.088)	0.876 (± 0.142)	0.858 (± 0.140)	0.351 (− 0.020 to 0.057)
EQ-VAS	63.8 (± 22.3)	62.2 (± 20.5)	0.592 (− 5.5 to 6.9)	79.4 (± 13.7)	74.4 (± 15.5)	0.055 (− 0.1 to 8.9)	81.2 (± 15.6)	79.4 (± 14.9)	0.405 (− 2.4 to 5.9)

*CIM* customised individually made, *OTS* off-the-shelf, *n* number*, SD* standard deviation, *CI* confidence interval, *KSS* Knee Society Score, *KOOS* Knee injury and Osteoarthritis Outcome Score, *FJS-12* Forgotten Joint Score*,* *VAS* visual analogue scale

The KSS after 4 and 12 months was higher for CIM TKA (*p* < 0.001, Table 2). Anatomical alignment and stability was better for CIM TKA (Table 3 and Supplementary material Table 6). For some patients (14%), the KSS after 12 months was missing: they did not attend the control visit because of freedom of complaints or restrictions due to the pandemic situation in 2020.

**Table 3:** Surgeon completed part of the KSS in detail before and after the surgery

KSS		Before surgery					12 months				
		CIM *n* = 74		OTS *n* = 169		*p* value	CIM *n* = 66		OTS *n* = 144		*p* value
Anatomic alignment, n (%)						0.150					0.072
	Neutral: 2–10° valgus	12	(16%)	42	(25%)		64	(97%)	130	(90%)	
	Varus: < 2° valgus	46	(62%)	83	(49%)		1	(2%)	13	(9%)	
	Valgus: > 10° valgus	16	(22%)	44	(26%)		1	(2%)	1	(1%)	
Medial/lateral instability, *n* (%)						< 0.001					< 0.001
	None	0	(0%)	19	(11%)		55	(83%)	71	(49%)	
	Little or < 5 mm	20	(27%)	95	(56%)		10	(15%)	60	(42%)	
	Moderate or 5 mm	45	(61%)	48	(28%)		1	(2%)	13	(9%)	
	Severe or > 5 mm	9	(12%)	7	(4%)		0	(0%)	0	(0%)	
Anterior/posterior instability, *n* (%)						< 0.001					< 0.001
	None	5	(7%)	56	(33%)		60	(91%)	76	(53%)	
	Moderate < 5 mm	64	(86%)	109	(65%)		6	(9%)	66	(46%)	
	Severe > 5 mm	5	(7%)	4	(2%)		0	(0%)	2	(1%)	
Range of motion, mean° (± SD)		118	(± 15)	116	(± 15)	0.268	126	(± 10)	124	(± 10)	0.378
Flexion contracture, *n* (%)						0.088					0.234
	None	18	(24%)	53	(31%)		64	(97%)	132	(92%)	
	1–5°	20	(27%)	55	(33%)		1	(2%)	12	(8%)	
	6–10°	24	(34%)	40	(24%)		0	(0%)	0	(0%)	
	11–15°	9	(11%)	19	(11%)		1	(2%)	0	(0%)	
	> 15°	3	(4%)	2	(1%)		0	(0%)	0	(0%)	
Extensor lag, *n* (%)						0.193					0.236
	None	18	(24%)	50	(30%)		64	(97%)	133	(92%)	
	< 10°	38	(51%)	93	(55%)		1	(2%)	11	(8%)	
	10–20°	17	(23%)	26	(15%)		1	(2%)	0	(0%)	
	> 20°	1	(1%)	0	(0%)		0	(0%)	0	(0%)	

*CIM* customised individually made, *OTS* off-the-shelf, *KSS* Knee Society Score, *n* number*, SD* standard deviation

**Fig. 2:**
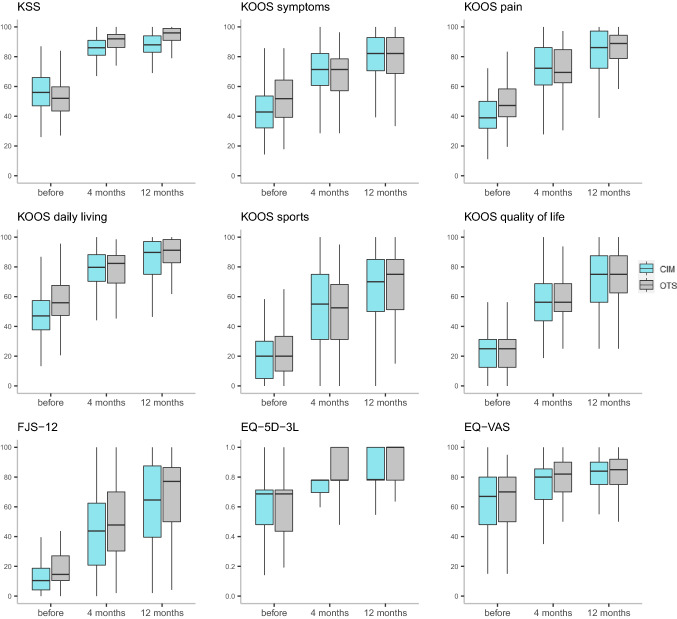
Boxplots of KSS and PROMs before and after the surgery. *CIM* customised individually made, *OTS* off-the-shelf, *KSS* Knee Society Score*, KOOS* Knee injury and Osteoarthritis Outcome Score, *FJS-12* Forgotten Joint Score*,* *VAS* visual analogue scale

**Fig. 3:**
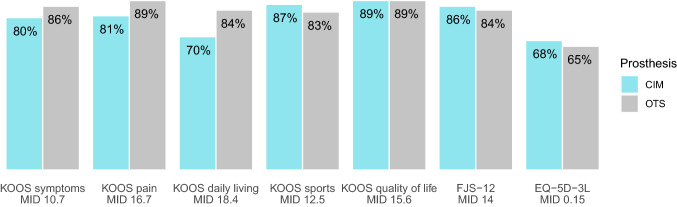
Proportion of patients with PROM results above the MID after 12 months. *CIM* customised individually made, *OTS* off-the-shelf, *MID* minimal important difference, *KOOS* Knee injury and Osteoarthritis Outcome Score, *FJS-12* Forgotten Joint Score

Patient satisfaction was high in both groups and did not differ after 4 and 12 months (*p* = 0.670 and *p* = 0.663, Table 4). All PROMs improved for both groups from baseline to 4 and 12 months, and from 4 to 12 months (*p* < 0.001). No differences in PROMs were found between the groups after 12 months (Table 2, Fig. 2). In both groups, the majority of patients improved above the MID after 12 months (Fig. 3). For overall improvement, most patients reported a “very much better” or “substantially better” overall knee state (CIM 86%, OTS 87%). Almost all patients would undergo the surgery again (CIM 95%, OTS 95%).

**Table 4:** Patient satisfaction after 4 and 12 months

	4 months				12 months			
	CIM *n* = 70		OTS *n* = 160		CIM *n* = 74		OTS *n* = 168	
Satisfied	60	(86%)	141	(88%)	64	(87%)	150	(89%)
Very satisfied	30	(43%)	77	(48%)	34	(46%)	78	(46%)
Satisfied	30	(43%)	64	(40%)	30	(41%)	72	(43%)
Not satisfied	10	(14%)	19	(12%)	10	(13%)	18	(11%)
Neutral	7	(10%)	18	(11%)	5	(7%)	15	(9%)
Unsatisfied	1	(1%)	1	(1%)	4	(5%)	3	(2%)
Very unsatisfied	2	(3%)	0	(0%)	1	(1%)	0	(0%)

*CIM* customised individually made, *OTS* off-the-shelf, *n* number

Adverse events are described in Table 5. Revision rate was 0% for CIM TKA and 2% (4/182) for OTS TKA.

**Table 5:** Number of postoperative adverse events

Adverse events	CIM	OTS
Arthrolysis	2	
Diagnostic arthroscopy to exclude an infection	1	
Pulmonary embolism		1
Deceased		1
Secondary patellar resurfacing		2*
Complete revision		2*
Quadriceps tendon rupture		1*

*CIM* customised individually made, *OTS* off-the-shelf, *n* number

*Excluded from the analyses

### Subgroup differences

Male patients were on average 1.6 years younger (p = 0.148) and mostly presented with higher preoperative PROMs, in total and in both TKA groups. Postoperative, no differences were found in PROMs compared to female patients (Supplementary material Table 8 and Fig. 4). Satisfaction after 12 months was slightly higher in female patients (female 90%, male 85%; *p* = 0.164) but not correlated to sex.

One third of the patients were younger than 65 years. Subgroup analyses between patients younger and older than 65 years revealed no preoperative differences. After 4 months, younger patients presented with lower PROMs (Supplementary material Table 9 and Fig. 5). After 12 months, the KOOS symptoms and KOOS quality of life were still lower for younger patients. Satisfaction after 12 months did not differ (younger 88%, older 89%; *p* = 1.000) and was not correlated to age.

### Correlation

Satisfaction after 12 months was not correlated to any variables of patients’ characteristics (*p* = 0.121 to 0.963), type of implant (*p* = 0.740), preoperative KSS (*p* = 0.446) or preoperative PROMs (*p* = 0.168 to 0.892). A medium to strong correlation existed between satisfaction after 12 months and all outcomes after 4 and 12 months (Supplementary material Table 6).

## Discussion

The most important finding of the present study was that patient satisfaction after 12 months was high and did not differ between CIM and OTS TKA. Our hypothesis could not be confirmed. Further, the percentage of satisfied patients in both groups was in the same range or slightly higher than reported in previous studies [6, 29, 47, 55].

Preoperative, patients in the CIM TKA group tend to be younger, male and presented with less subjective impairment. In our hospital, CIM TKA are currently only covered for patients with a supplementary insurance, which explains the difference in insurance status and might be an indicator for a higher socio-economic status. However, data about the socio-economic status of our patients are not available.

The current study is the first to prospectively compare CIM and OTS TKA regarding patient satisfaction and PROMs. A high satisfaction rate and clear improvements over time in all PROMs were found; however, there were no differences between both groups. Another retrospective study found comparable KOOS results, also without any differences, but with a higher satisfaction in patients with CIM TKA [32]. The results of the KOOS sports were lower compared to the other KOOS subscales, which can possibly be explained by the advanced age at which patients no longer perform activities like running, jumping or kneeling. Advanced PROMs, like the FJS-12, are more discriminating and might be more appropriate for younger and active patients [49]. Patients with CIM TKA tended to have a higher FJS-12, indicating that these patients are less aware of their artificial joint in everyday life. However, the FJS-12 was also noticeably lower in younger patients.

The postoperative KSS was high and, likewise in another study [32], higher for CIM TKA. CIM TKA were mostly neutrally aligned, which is due to the implant design, instruments and the planning of the surgery [45]. Stability was clearly better in CIM TKA, notably the issue of midflexion instability was eliminated in most of the cases [33, 43]. The shape of the CIM implant resembles the original condylar shapes, consequently, the interaction between the individually given ligaments and the implanted prosthesis results in better kinematical behaviour. It has been shown, that CIM TKAs are associated with an increased manipulation rate due to sporadic lower range of motion [53], which we could not confirm. Understandably, surgeons tend to fit CIM TKA too tight in the beginning, because of the initial fear to resect too much bone and due to the limited implant options. Experience helps to find the ideal laxity references intraoperative which results in a low manipulation rate.

A higher rate of revisions and adverse events was observed in patients with OTS TKA. It might be due to the smaller sample of CIM TKA. A longer follow-up is needed to clearly show differences between the two groups. Regardless, our findings are in accordance with data from the Swiss Implant Registry: the 2-year revision rate is 3.4% (iTotal: data not yet available; Attune^®^: 3.6%), with patellar problems occurring in about every third patient scheduled for revision [42].

Other studies on CIM TKA found improved alignment [18, 36, 45], decreased blood loss and hospital stay [39] and reduced total costs [27]. A large retrospective study recently found a high satisfaction rate (89%, mean follow-up 2.8 years) and high implant survivorship (98.5%) [37]. However, based on the current evidence, CIM TKA are not clearly superior [24, 25, 51] and long-term results are not yet available. A recent systematic review revealed the lack of strong methodological studies [51]. Dissatisfaction after TKA remains challenging and influencing factors are not yet fully understood. In addition to implant design and clinical outcome, PROMs need to be further investigated.

Strengths of this study are the prospective comparative design and the number of recruited patients, as the availability of CIM TKAs is relatively recent. The number of loss to follow-up was very low and a broad set of PROMs was used. Nevertheless, our study has some limitations and needs to be interpreted accordingly. A selection bias could not be prevented as we are located in a private hospital and CIM TKA patients require a supplementary insurance. Given that and the possible intolerance of patients, a randomised controlled trial has not yet been practical. However, the Attune^®^ implant in the OTS TKA group is with over 3000 implants per year by far the most applied implant in Switzerland [42]. Because of the observational study design, the influence of other confounders cannot be excluded. The study focussed on patient satisfaction and PROMs, hence, knee alignment was not examined in detail, e.g. the hip–knee–ankle angle. Relevant differences between the two groups in postoperative alignment, classified according to the KSS, were not found. The follow-up is only short-term as long-term data are preferable but not yet available. However, for studies with PROMs as primary outcome, it was shown that a 12-month follow-up after TKA is adequate as results are consistent [28, 35]. This study showed that patient satisfaction is high for CIM and OTS TKA, and both achieve good clinical outcome and PROMs.

## Conclusions

This prospective cohort study found no difference in satisfaction between patients with CIM and OTS TKA after 12 months. In both groups, satisfaction was high and PROMs improved considerably. We believe that it is important to further investigate patient-reported outcome and factors that affect the outcome to gain a better understanding of satisfaction after TKA.

## Supplementary Information

Below is the link to the electronic supplementary material.

Supplementary file1 (DOCX 39 KB)

Supplementary file2 Boxplots of KSS and PROMs before and after the surgery: comparison female and male patients. KSS Knee Society Score, KOOS Knee injury and Osteoarthritis Outcome Score, FJS-12 Forgotten Joint Score, VAS visual analogue scale (PDF 15 KB)

Supplementary file3 Boxplots of KSS and PROMs before and after the surgery: comparison younger and older patients. KSS Knee Society Score, KOOS Knee injury and Osteoarthritis Outcome Score, FJS-12 Forgotten Joint Score, VAS visual analogue scale (PDF 15 KB)

## Abbreviations

ASA: American Society of Anesthesiologists
CIM: Customised individually made
FDA: US Food and Drug Administration
FJS-12: Forgotten Joint Score
KOOS: Knee injury and Osteoarthritis Outcome Score
KSS: Knee Society Score
MID: Minimal important difference
OTS: Off-the-shelf
PROM: Patient-reported outcome measure
SD: Standard deviation
TKA: Total knee arthroplasty
VAS: Visual analogue scale

## References

[1] American Society of Anesthesiologists (2021) ASA physical status classification system. https://www.asahq.org/resources/clinical-information/asa-physical-status-classification-system Accessed 14 Jan 2021

[2] Arbab D, Reimann P, Brucker M, Bouillon B, Lüring C (2018). Alignment in total knee arthroplasty—a comparison of patient-specific implants with the conventional technique. Knee.

[3] Baker PN, van der Meulen JH, Lewsey J, Gregg PJ (2007). The role of pain and function in determining patient satisfaction after total knee replacement. Data from the National Joint Registry for England and Wales. J Bone Jt Surg Br.

[4] Beckers L, Müller JH, Daxhelet J, Ratano S, Saffarini M, Aït-Si-Selmi T, Bonnin MP (2021). Considerable inter-individual variability of tibial geometric ratios renders bone-implant mismatch unavoidable using off-the-shelf total knee arthroplasty: a systematic review and meta-analysis. Knee Surg Sports Traumatol Arthrosc.

[5] Beckmann J, Meier MK, Benignus C, Hecker A, Thienpont E (2021). Contemporary knee arthroplasty: one fits all or time for diversity?. Arch Orthop Trauma Surg.

[6] Bourne RB, Chesworth BM, Davis AM, Mahomed NN, Charron KDJ (2010). Patient satisfaction after total knee arthroplasty: who is satisfied and who is not?. Clin Orthop.

[7] Budhiparama NC, Lumban-Gaol I, Ifran NN, de Groot PCJ, Utomo DN, Nelissen RGHH (2021). Mismatched knee implants in Indonesian and Dutch patients: a need for increasing the size. Knee Surg Sports Traumatol Arthrosc.

[8] Cohen J (1988). Statistical power analysis for the behavioral sciences.

[9] Conformis (2021) https://www.conformis.com/about-conformis/news/conformis-receives-ce-mark-certification-for-itotal-cr-patient-specific-total-knee-resurfacing-system/ Accessed 10 Oct 2021

[10] Devji T, Guyatt GH, Lytvyn L, Brignardello-Petersen R, Foroutan F, Sadeghirad B, Buchbinder R, Poolman RW, Harris IA, Carrasco-Labra A, Siemieniuk RAC, Vandvik PO (2017). Application of minimal important differences in degenerative knee disease outcomes: a systematic review and case study to inform BMJ rapid recommendations. BMJ Open.

[11] EQ-5D (2021) https://euroqol.org/eq-5d-instruments/eq-5d-3l-about/ Accessed 14 Jan 2021

[12] Gunaratne R, Pratt DN, Banda J, Fick DP, Khan RJK, Robertson BW (2017). Patient dissatisfaction following total knee arthroplasty: a systematic review of the literature. J Arthroplasty.

[13] Hirschmann MT, Moser LB, Amsler F, Behrend H, Leclerq V, Hess S (2019). Functional knee phenotypes: a novel classification for phenotyping the coronal lower limb alignment based on the native alignment in young non-osteoarthritic patients. Knee Surg Sports Traumatol Arthrosc.

[14] Holtz N, Hamilton DF, Giesinger JM, Jost B, Giesinger K (2020). Minimal important differences for the WOMAC osteoarthritis index and the Forgotten Joint Score-12 in total knee arthroplasty patients. BMC Musculoskelet Disord.

[15] Kahlenberg CA, Nwachukwu BU, McLawhorn AS, Cross MB, Cornell CN, Padgett DE (2018). Patient satisfaction after total knee replacement: a systematic review. HSS J.

[16] Kohn MD, Sassoon AA, Fernando ND (2016). Classifications in brief: Kellgren–Lawrence classification of osteoarthritis. Clin Orthop Relat Res.

[17] Lee J-A, Koh Y-G, Kang K-T (2020). Biomechanical and clinical effect of patient-specific or customized knee implants: a review. J Clin Med.

[18] Levengood GA, Dupee J (2018). Accuracy of coronal plane mechanical alignment in a customized, individually made total knee replacement with patient-specific instrumentation. J Knee Surg.

[19] Li K, Saffarini M, Valluy J, Desseroit M-C, Morvan Y, Telmon N, Cavaignac E (2019). Sexual and ethnic polymorphism render prosthetic overhang and under-coverage inevitable using off-the shelf TKA implants. Knee Surg Sports Traumatol Arthrosc.

[20] Mahoney OM, Kinsey T (2010). Overhang of the femoral component in total knee arthroplasty: risk factors and clinical consequences. J Bone Jt Surg Am.

[21] Maratt JD, Lee Y, Lyman S, Westrich GH (2015). Predictors of satisfaction following total knee arthroplasty. J Arthroplasty.

[22] Meier M, Janssen D, Koeck FX, Thienpont E, Beckmann J, Best R (2021). Variations in medial and lateral slope and medial proximal tibial angle. Knee Surg Sports Traumatol Arthrosc.

[23] Monticone M, Ferrante S, Salvaderi S, Motta L, Cerri C (2013). Responsiveness and minimal important changes for the Knee Injury and Osteoarthritis Outcome Score in subjects undergoing rehabilitation after total knee arthroplasty. Am J Phys Med Rehabil.

[24] Moret CS, Schelker BL, Hirschmann MT (2021). Clinical and radiological outcomes after knee arthroplasty with patient-specific versus off-the-shelf knee implants: a systematic review. J Pers Med.

[25] Müller JH, Liebensteiner M, Kort N, Stirling P, Pilot P, Demey G, European Knee Associates (EKA) (2021). No significant difference in early clinical outcomes of custom versus off-the-shelf total knee arthroplasty: a systematic review and meta-analysis. Knee Surg Sports Traumatol Arthrosc.

[26] Noble PC, Gordon MJ, Weiss JM, Reddix RN, Conditt MA, Mathis KB (2005). Does total knee replacement restore normal knee function?. Clin Orthop Relat Res.

[27] O’Connor MI, Blau BE (2019). The economic value of customized versus off-the-shelf knee implants in medicare fee-for-service beneficiaries. Am Health Drug Benefits.

[28] Piuzzi NS, Cleveland Clinic O. M. E. Arthroplasty Group (2021). Patient-reported outcomes at 1 and 2 years after total hip and knee arthroplasty: what is the minimum required follow-up?. Arch Orthop Trauma Surg.

[29] Pronk Y, Peters MCWM, Brinkman J-M (2021). Is patient satisfaction after total knee arthroplasty predictable using patient characteristics and preoperative patient-reported outcomes?. J Arthroplasty.

[30] R Core Team (2021). R: a language and environment for statistical computing.

[31] Ravi B, Croxford R, Reichmann WM, Losina E, Katz JN, Hawker GA (2012). The changing demographics of total joint arthroplasty recipients in the United States and Ontario from 2001 to 2007. Best Pract Res Clin Rheumatol.

[32] Reimann P, Brucker M, Arbab D, Lüring C (2019). Patient satisfaction—a comparison between patient-specific implants and conventional total knee arthroplasty. J Orthop.

[33] Romero J, Duronio JF, Sohrabi A, Alexander N, MacWilliams BA, Jones LC, Hungerford DS (2002). Varus and valgus flexion laxity of total knee alignment methods in loaded cadaveric knees. Clin Orthop Relat Res.

[34] Roos EM, Lohmander LS (2003). The knee injury and osteoarthritis outcome score (KOOS): from joint injury to osteoarthritis. Health Qual Life Outcomes.

[35] Schoenmakers DAL, Schotanus MGM, Boonen B, Kort NP (2018). Consistency in patient-reported outcome measures after total knee arthroplasty using patient-specific instrumentation: a 5-year follow-up of 200 consecutive cases. Knee Surg Sports Traumatol Arthrosc.

[36] Schroeder L, Martin G (2019). In vivo tibial fit and rotational analysis of a customized, patient-specific TKA versus off-the-shelf TKA. J Knee Surg.

[37] Schroeder L, Pumilia CA, Sarpong NO, Martin G (2021). Patient satisfaction, functional outcomes, and implant survivorship in patients undergoing customized cruciate-retaining TKA. JBJS Rev.

[38] Schünemann HJ, Guyatt GH (2005). Commentary–goodbye M(C)ID! Hello MID, where do you come from?. Health Serv Res.

[39] Schwarzkopf R, Brodsky M, Garcia GA, Gomoll AH (2015). Surgical and functional outcomes in patients undergoing total knee replacement with patient-specific implants compared with “off-the-shelf” implants. Orthop J Sports Med.

[40] Scuderi GR, Bourne RB, Noble PC, Benjamin JB, Lonner JH, Scott WN (2012). The new knee society knee scoring system. Clin Orthop Relat Res.

[41] Simsek ME, Akkaya M, Gursoy S, Isik C, Zahar A, Tarabichi S, Bozkurt M (2018). Posterolateral overhang affects patient quality of life after total knee arthroplasty. Arch Orthop Trauma Surg.

[42] SIRIS. Report Hip & Knee (2021) http://www.siris-implant.ch/de/Downloads&amp;category=16 Accessed 24 Sept 2021

[43] Stähelin T, Kessler O, Pfirrmann C, Jacob HAC, Romero J (2003). Fluoroscopically assisted stress radiography for varus-valgus stability assessment in flexion after total knee arthroplasty. J Arthroplasty.

[44] Steinert AF, Sefrin L, Hoberg M, Arnholdt J, Rudert M (2015). Individualendoprothetik am Kniegelenk. Orthopäde.

[45] Steinert AF, Sefrin L, Jansen B, Schröder L, Holzapfel BM, Arnholdt J, Rudert M (2021). Patient-specific cruciate-retaining total knee replacement with individualized implants and instruments (iTotal^TM^ CR G2). Oper Orthop Traumatol.

[46] Swissethics. Registry of all Projects in Switzerland (2021) https://ongoingprojects.swissethics.ch/runningProjects_list.php?q=%28BASECID~contains~2016-01777%29&orderby=dBASECID Accessed 27 Jul 2021

[47] The Swedish Knee Arthroplasty Register (2021) Annual Report 2020. https://www.myknee.se/en/ Accessed 10 Oct 2021

[48] The World Medical Association (2021). Declaration of Helsinki – Ethical Principles for Medical Research Involving Human Subjects. https://www.wma.net/policies-post/wma-declaration-of-helsinki-ethical-principles-for-medical-research-involving-human-subjects/ Accessed 14 Sept 2021

[49] Thompson SM, Salmon LJ, Webb JM, Pinczewski LA, Roe JP (2015). Construct validity and test re-test reliability of the forgotten joint score. J Arthroplasty.

[50] Thomsen MG, Latifi R, Kallemose T, Barfod KW, Husted H, Troelsen A (2016). Good validity and reliability of the forgotten joint score in evaluating the outcome of total knee arthroplasty. Acta Orthop.

[51] Victor J, Vermue H (2021). Custom TKA: what to expect and where do we stand today?. Arch Orthop Trauma Surg.

[52] Vogel N, Rychen T, Kaelin R, Arnold MP (2020). Patient-reported outcome measures (PROMs) following knee arthroplasty: a prospective cohort study protocol. BMJ Open.

[53] White PB, Ranawat AS (2016). Patient-specific total knees demonstrate a higher manipulation rate compared to “off-the-shelf implants”. J Arthroplasty.

[54] Wunderlich F, Azad M, Westphal R, Klonschinski T, Belikan P, Drees P, Eckhard L (2021). Comparison of postoperative coronal leg alignment in customized individually made and conventional total Knee arthroplasty. J Pers Med.

[55] Young-Shand KL, Dunbar MJ, Laende EK, Mills Flemming JE, Astephen Wilson JL (2021). Early identification of patient satisfaction two years after total knee arthroplasty. J Arthroplasty.

