# Supplementary material for: No difference in patient-reported satisfaction after 12 months between customised individually made and off-the-shelf total knee arthroplasty
Source: Knee Surg Sports Traumatol Arthrosc. 2022 Feb 12;30(9):2948–57. doi: 10.1007/s00167-022-06900-z (PMC9418302; doi:10.1007/s00167-022-06900-z)
Supplement: Supplementary file 1 — Supplementary file1 (DOCX 39 KB) [file 167_2022_6900_MOESM1_ESM.docx]

# Supplementary material

## Table 6

Correlation between satisfaction after 12 months and outcomes after 4 and 12 months

|  | | | **Satisfaction 12 months** | | | | | | |
| --- | --- | --- | --- | --- | --- | --- | --- | --- | --- |
|  |  |  | **CIM** | | |  | **OTS** | | |
|  |  |  | Spearman r | P value | Correlation |  | Spearman r | P value | Correlation |
| **4 months outcomes** | | | | | | | | | |
|  | KSS |  | 0.465 | < 0.001 | strong |  | 0.281 | < 0.001 | medium |
|  | KOOS symptoms |  | 0.563 | < 0.001 | strong |  | 0.375 | < 0.001 | medium |
|  | KOOS pain |  | 0.612 | < 0.001 | strong |  | 0.369 | < 0.001 | medium |
|  | KOOS daily living |  | 0.686 | < 0.001 | strong |  | 0.429 | < 0.001 | strong |
|  | KOOS sports |  | 0.525 | < 0.001 | strong |  | 0.352 | < 0.001 | medium |
|  | KOOS quality of life |  | 0.601 | < 0.001 | strong |  | 0.346 | < 0.001 | medium |
|  | FJS-12 |  | 0.514 | < 0.001 | strong |  | 0.367 | < 0.001 | medium |
|  | EQ-5D-3L |  | 0.481 | < 0.001 | strong |  | 0.302 | < 0.001 | medium |
|  | EQ-5D VAS |  | 0.388 | 0.001 | medium |  | 0.265 | 0.001 | medium |
| **12 months outcomes** | | | | | | | | | |
|  | KSS |  | 0.720 | < 0.001 | strong |  | 0.312 | < 0.001 | medium |
|  | KOOS symptoms |  | 0.651 | < 0.001 | strong |  | 0.625 | < 0.001 | strong |
|  | KOOS pain |  | 0.721 | < 0.001 | strong |  | 0.669 | < 0.001 | strong |
|  | KOOS daily living |  | 0.731 | < 0.001 | strong |  | 0.632 | < 0.001 | strong |
|  | KOOS sports |  | 0.672 | < 0.001 | strong |  | 0.557 | < 0.001 | strong |
|  | KOOS quality of life |  | 0.730 | < 0.001 | strong |  | 0.647 | < 0.001 | strong |
|  | FJS-12 |  | 0.665 | < 0.001 | strong |  | 0.627 | < 0.001 | strong |
|  | EQ-5D-3L |  | 0.652 | < 0.001 | strong |  | 0.377 | < 0.001 | medium |
|  | EQ-5D VAS |  | 0.473 | < 0.001 | strong |  | 0.303 | < 0.001 | medium |

*CIM* customised individually made, *OTS* off-the-shelf, *KSS* Knee Society Score, *KOOS* Knee injury and Osteoarthritis Outcome Score, *FJS-12* Forgotten Joint Score*,* VAS visual analogue scale

## Table 7

Surgeon completed part of the KSS in detail before and after the surgery.

| **KSS** | | **Before surgery** | | | | |  | **4 months** | | | | |  | **12 months** | | | | |
| --- | --- | --- | --- | --- | --- | --- | --- | --- | --- | --- | --- | --- | --- | --- | --- | --- | --- | --- |
|  |  | CIM  n = 74 | | OTS  n = 169 | | P value |  | CIM  n = 73 | | OTS  n = 165 | | P value |  | CIM  n = 66 | | OTS  n = 144 | | P value |
| Anatomic alignment**,** n (%) | |  |  |  |  | 0.150 |  |  |  |  |  | 0.028 |  |  |  |  |  | 0.072 |
|  | Neutral: 2 to 10° valgus | 12 | (16%) | 42 | (24%) |  |  | 71 | (97%) | 143 | (87%) |  |  | 64 | (97%) | 130 | (90%) |  |
|  | Varus: < 2° valgus | 46 | (62%) | 83 | (49%) |  |  | 1 | (1%) | 17 | (10%) |  |  | 1 | (2%) | 13 | (9%) |  |
|  | Valgus: > 10° valgus | 16 | (22%) | 44 | (26%) |  |  | 1 | (1%) | 5 | (3%) |  |  | 1 | (2%) | 1 | (1%) |  |
| Medial / Lateral instability**,** n (%) | | | | | | <0.001 |  |  |  |  |  | <0.001 |  |  |  |  |  | <0.001 |
|  | None | 0 | (0%) | 19 | (11%) |  |  | 62 | (85%) | 81 | (49%) |  |  | 55 | (83%) | 71 | (49%) |  |
|  | Little or < 5 mm | 20 | (27%) | 95 | (56%) |  |  | 10 | (14%) | 75 | (45%) |  |  | 10 | (15%) | 60 | (42%) |  |
|  | Moderate or 5 mm | 45 | (61%) | 48 | (28%) |  |  | 1 | (1%) | 9 | (6%) |  |  | 1 | (2%) | 13 | (9%) |  |
|  | Severe or > 5 mm | 9 | (12%) | 7 | (4%) |  |  | 0 | (0%) | 0 | (0%) |  |  | 0 | (0%) | 0 | (0%) |  |
| Anterior / Posterior instability**,** n (%) | | | | | | <0.001 |  |  |  |  |  | <0.001 |  |  |  |  |  | <0.001 |
|  | None | 5 | (7%) | 56 | (33%) |  |  | 68 | (93%) | 90 | (54%) |  |  | 60 | (91%) | 76 | (53%) |  |
|  | Moderate < 5 mm | 64 | (86%) | 109 | (65%) |  |  | 5 | (7%) | 74 | (45%) |  |  | 6 | (9%) | 66 | (46%) |  |
|  | Severe > 5 mm | 5 | (7%) | 4 | (2%) |  |  | 0 | (0%) | 1 | (1%) |  |  | 0 | (0%) | 2 | (1%) |  |
| Range of motion, mean° (SD) | | 118 | (± 15) | 116 | (± 15) | 0.268 |  | 123 | (± 11) | 121 | (± 10) | 0.166 |  | 126 | (± 10) | 124 | (± 10) | 0.378 |
| Flexion contracture**,** n (%) | |  |  |  |  | 0.107 |  |  |  |  |  | 0.694 |  |  |  |  |  | 0.234 |
|  | None | 18 | (23%) | 53 | (31%) |  |  | 62 | (85%) | 143 | (87%) |  |  | 64 | (97%) | 132 | (92%) |  |
|  | 1 to 5° | 20 | (27%) | 55 | (33%) |  |  | 9 | (13%) | 19 | (12%) |  |  | 1 | (2%) | 12 | (8%) |  |
|  | 6 to 10° | 24 | (34%) | 40 | (24%) |  |  | 1 | (1%) | 2 | (1%) |  |  | 0 | (0%) | 0 | (0%) |  |
|  | 11 to 15° | 9 | (11%) | 19 | (11%) |  |  | 1 | (1%) | 0 | (0%) |  |  | 1 | (2%) | 0 | (0%) |  |
|  | > 15° | 3 | (4%) | 2 | (1%) |  |  | 0 | (0%) | 1 | (1%) |  |  | 0 | (0%) | 0 | (0%) |  |
| Extensor lag**,** n (%) | |  |  |  |  | 0.193 |  |  |  |  |  | 0.478 |  |  |  |  |  | 0.236 |
|  | None | 18 | (24%) | 50 | (30%) |  |  | 61 | (83%) | 143 | (87%) |  |  | 64 | (97%) | 133 | (92%) |  |
|  | <10° | 38 | (51%) | 93 | (55%) |  |  | 10 | (14%) | 20 | (12%) |  |  | 1 | (2%) | 11 | (8%) |  |
|  | 10 to 20° | 17 | (23%) | 26 | (15%) |  |  | 2 | (3%) | 1 | (1%) |  |  | 1 | (2%) | 0 | (0%) |  |
|  | > 20° | 1 | (1%) | 0 | (0%) |  |  | 0 | (0%) | 1 | (1%) |  |  | 0 | (0%) | 0 | (0%) |  |

*CIM* customised individually made, *OTS* off-the-shelf, *KSS* Knee Society Score, *n* number*, SD* standard deviation

## Table 8

KSS and PROMs before and after the surgery: comparison male and female patients.

|  | **Before surgery** | | |  | **4 months** | | |  | **12 months** | | |
| --- | --- | --- | --- | --- | --- | --- | --- | --- | --- | --- | --- |
|  | Male  n = 109 | Female  n = 134 | P value |  | Male  n = 109 | Female  n = 134 | P value |  | Male  n = 109 | Female  n = 134 | P value |
|  | mean (± SD) | | (95% CI) |  | mean (± SD) | | (95% CI) |  | mean (± SD) | | (95% CI) |
| **KSS** | 57.0  (± 13.1) | 54.2  (± 13.0) | 0.174  (-1.0 to 5.6) |  | 87.1  (± 9.6) | 86.6  (± 8.5) | 0.695  (-1.9 to 2.8) |  | 91.0  (± 8.9) | 90.1  (± 8.4) | 0.421  (-1.4 to 3.3) |
| **KOOS symptoms** | 52.4  (± 19.1) | 43.6  (± 15.5) | < 0.001  (4.5 to 13.3) |  | 68.5  (± 16.5) | 70.3  (± 15.7) | 0.394  (-5.9 to 2.3) |  | 79.3  (± 14.9) | 78.4  (± 15.4) | 0.645  (-2.9 to 4.8) |
| **KOOS**  **pain** | 48.1  (± 16.4) | 41.0  (± 12.9) | < 0.001  (3.4 to 10.8) |  | 72.1  (± 16.9) | 71.3  (± 16.7) | 0.746  (-3.6 to 5.0) |  | 83.3  (± 15.2) | 82.6  (± 16.4) | 0.764  (-3.4 to 4.7) |
| **KOOS**  **daily living** | 54.7  (± 16.4) | 47.9  (± 15.6) | 0.001  (2.6 to 10.8) |  | 77.5  (± 14.8) | 77.3  (± 13.7) | 0.875  (-3.4 to 3.9) |  | 85.6  (± 14.5) | 84.9  (± 14.2) | 0.707  (-2.9to 4.3) |
| **KOOS**  **sports** | 25.4  (± 17.9) | 17.3  (± 18.2) | < 0.001  (23.3 to 12.9) |  | 54.4  (± 24.4) | 47.2  (± 26.4) | 0.062  (-0.4 to 14.8) |  | 65.9  (± 22.9) | 62.5  (± 28.2) | 0.328  (-3.5 to 10.4) |
| **KOOS**  **quality of life** | 27.0  (± 13.1) | 22.8  (± 13.9) | 0.016  (0.8 to 7.8) |  | 57.2  (± 22.0) | 58.6  (± 19.0) | 0.598  (-6.6 to 3.8) |  | 71.1  (± 21.1) | 69.8  (± 22.2) | 0.647  (-4.2 to 6.8) |
| **FJS-12** | 17.3  (± 14.9) | 12.6  (± 10.6) | 0.006  (1.5 to 8.0) |  | 44.0  (± 28.2) | 43.4  (± 24.2) | 0.864  (-6.2 to 7.3) |  | 64.1  (± 26.8) | 60.6  (± 29.1) | 0.345  (-3.7 to 10.6) |
| **EQ-5D-3L** | 0.639  (± 0.179) | 0.594  (± 0.182) | 0.104  (-0.008 to 0.090) |  | 0.803  (± 0.173) | 0.785  (± 0.133) | 0.334  (-0.022 to 0.064) |  | 0.875  (± 0.141) | 0.855  (± 0.139) | 0.279  (-0.017 to 0.058) |
| **EQ-VAS** | 60.6  (± 23.0) | 64.4  (± 19.2) | 0.181  (-9.3 to 1.7) |  | 74.5  (± 16.2) | 77.0  (± 14.0) | 0.207  (-6.5 to 1.4) |  | 80.8  (± 15.7) | 79.3  (± 14.6) | 0.429  (-2.3 to 5.4) |

*CIM* customised individually made, *OTS* off-the-shelf, *n* number*, SD* standard deviation, *CI* confidence interval, *KSS* Knee Society Score, *KOOS* Knee injury and Osteoarthritis Outcome Score, *FJS-12* Forgotten Joint Score*,* VAS visual analogue scale

## Table 9

KSS and PROMs before and after the surgery: comparison younger and older patients.

|  | **Before surgery** | | |  | **4 months** | | |  | **12 months** | | |
| --- | --- | --- | --- | --- | --- | --- | --- | --- | --- | --- | --- |
|  | < 65 years  n = 77 | ≥ 65 years  n = 166 | P value |  | < 65 years  n = 77 | ≥ 65 years  n = 166 | P value |  | < 65 years  n = 77 | ≥ 65 years  n = 166 | P value |
|  | mean (± SD) | | (95% CI) |  | mean (± SD) | | (95% CI) |  | mean (± SD) | | (95% CI) |
| **KSS** | 57.8  (± 14.1) | 54.8  (± 12.5) | 0.105  (-0.6 to 6.5) |  | 86.3  (± 9.5) | 87.1  (± 8.8) | 0.506  (-3.4 to 1.7) |  | 90.4  (± 9.4) | 90.7  (± 8.2) | 0.817  (-2.8 to 2.2) |
| **KOOS**  **symptoms** | 44.9  (± 20.4) | 48.8  (± 16.3) | 0.141  (-9.2 to 1.3) |  | 64.6  (± 15.2) | 71.7  (± 16.0) | 0.002  (-11.4 to -2.7) |  | 75.9  (± 15.3) | 80.1  (± 15.0) | 0.044  (-8.3 to -0.1) |
| **KOOS**  **pain** | 43.0  (± 16.8) | 44.8  (± 14.1) | 0.426  (-6.2 to 2.6) |  | 67.0  (± 16.2) | 73.8  (± 16.7) | 0.004  (-11.4 to -2.2) |  | 81.2  (± 15.9) | 83.7  (± 15.8) | 0.265  (-6.8 to 1.9) |
| **KOOS**  **daily living** | 50.9  (± 19.2) | 51.0  (± 14.8) | 0.949  (-5.1 to 4.8) |  | 74.5  (± 16.0) | 78.7  (± 13.2) | 0.034  (-8.1 to -0.3) |  | 84.5  (± 15.2) | 85.5  (± 13.9) | 0.612  (-4.9 to 2.9) |
| **KOOS**  **sports** | 19.0  (± 18.6) | 21.9  (± 18.4) | 0.277  (-8.0 to 2.3) |  | 46.1  (± 25.9) | 53.2  (± 24.6) | 0.065  (-14.7 to 0.4) |  | 61.5  (± 26.0) | 65.5  (± 25.7) | 0.282  (-11.3 to 3.3) |
| **KOOS**  **quality of life** | 22.5  (± 14.9) | 25.7  (± 13.1) | 0.087  (-7.0 to 0.5) |  | 51.4  (± 19.3) | 60.9  (± 20.2) | < 0.001  (-15.0 to -4.0) |  | 66.1  (± 22.4) | 72.5  (± 21.1) | 0.033  (-12.2 to -0.5) |
| **FJS-12** | 13.8  (± 13.2) | 15.1  (± 12.9) | 0.433  (-5.0 to 2.1) |  | 36.8  (± 26.6) | 46.7  (± 25.2) | 0.006  (-17.1 to -2.8) |  | 58.7  (± 28.2) | 63.8  (± 28.0) | 0.189  (-12.8 to 2.5) |
| **EQ-5D-3L** | 0.596  (± 0.208) | 0.622  (± 0.169) | 0.325  (-0.086 to 0.029) |  | 0.748  (± 0.166) | 0.814  (± 0.141) | 0.002  (-0.108 to  -0.024) |  | 0.853  (± 0.149) | 0.869  (± 0.137) | 0.407  (-0.055 to 0.022) |
| **EQ-VAS** | 63.8  (± 22.3) | 62.2  (± 20.5) | 0.592  (-4.3 to 7.5) |  | 79.4  (± 13.7) | 74.4  (± 15.4) | 0.021  (0.8 to 9.3) |  | 81.2  (± 15.6) | 79.4  (± 14.9) | 0.405  (-2.4 to 6.0) |

*CIM* customised individually made, *OTS* off-the-shelf, *n* number*, SD* standard deviation, *CI* confidence interval, *KSS* Knee Society Score, *KOOS* Knee injury and Osteoarthritis Outcome Score, *FJS-12* Forgotten Joint Score*,* VAS visual analogue scale
