# Supplementary figures and images for: No difference in patient-reported satisfaction after 12 months between customised individually made and off-the-shelf total knee arthroplasty
Source: Knee Surg Sports Traumatol Arthrosc. 2022 Feb 12;30(9):2948–57. doi: 10.1007/s00167-022-06900-z (PMC9418302; doi:10.1007/s00167-022-06900-z)

KSS

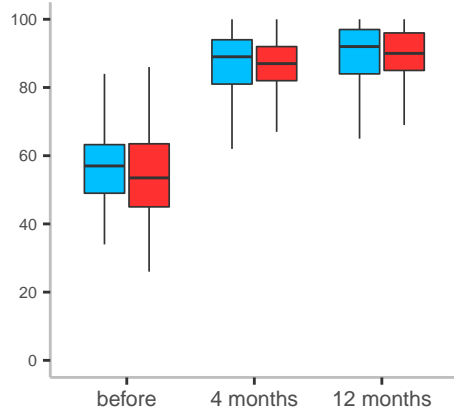

KOOS symptoms

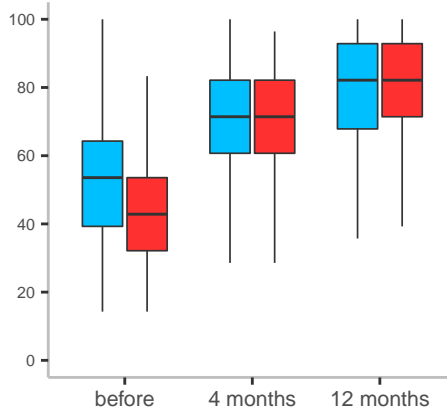

KOOS pain

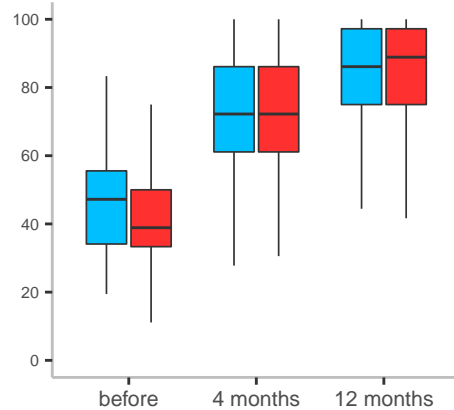

KOOS daily living

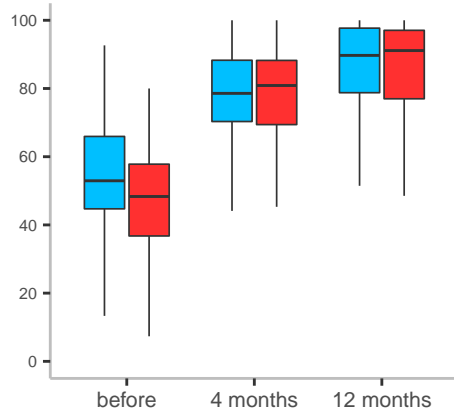

KOOS sports

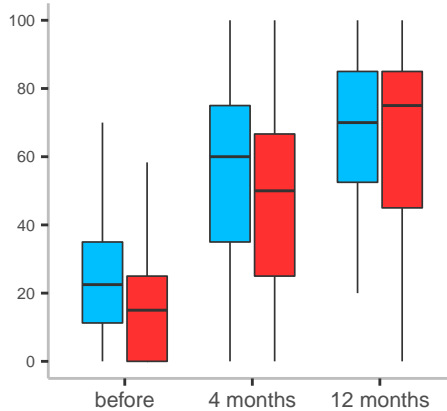

KOOS quality of life

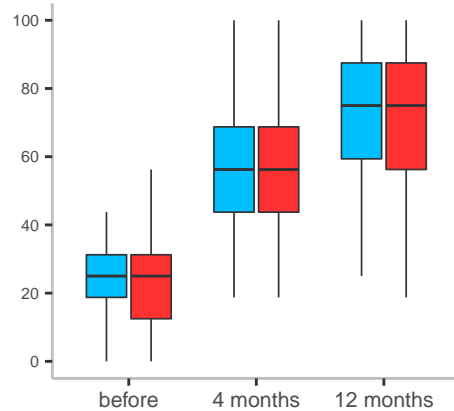

FJS-12

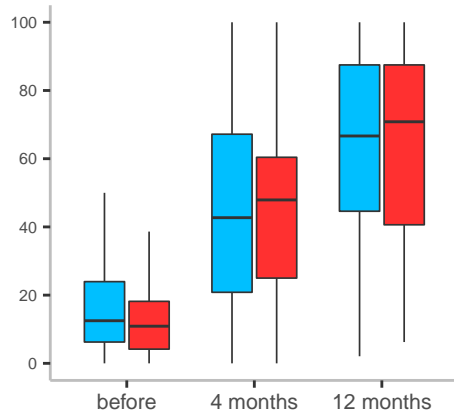

EQ-5D-3L

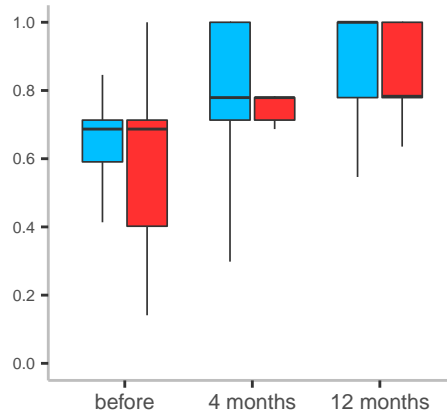

EQ-VAS

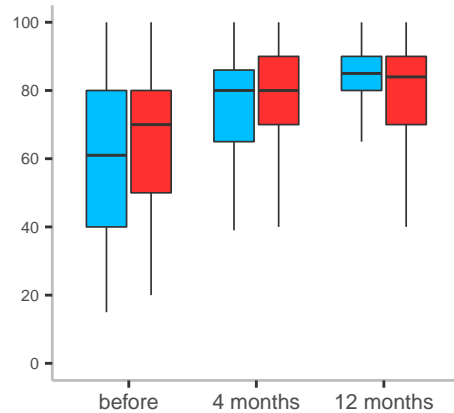

Supplement: Supplementary file 2 — Supplementary file2 Boxplots of KSS and PROMs before and after the surgery: comparison female and male patients. KSS Knee Society Score, KOOS Knee injury and Osteoarthritis Outcome Score, FJS-12 Forgotten Joint Score, VAS visual analogue scale (PDF 15 KB) [file 167_2022_6900_MOESM2_ESM.pdf]

KSS

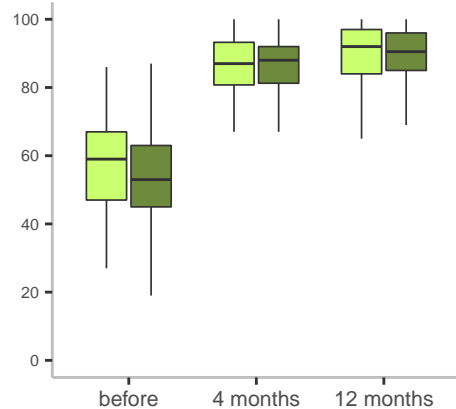

KOOS symptoms

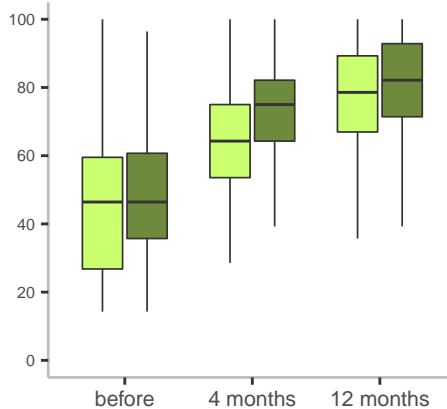

KOOS pain

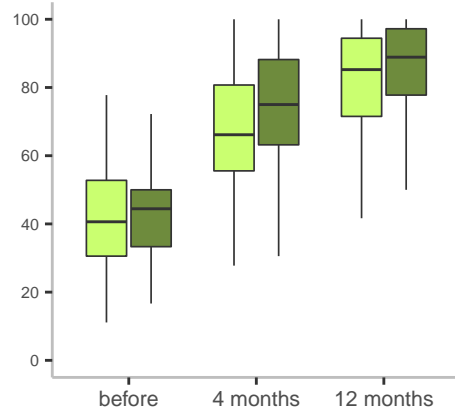

KOOS daily living

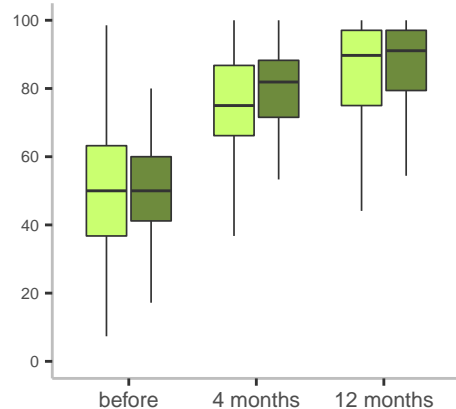

KOOS sports

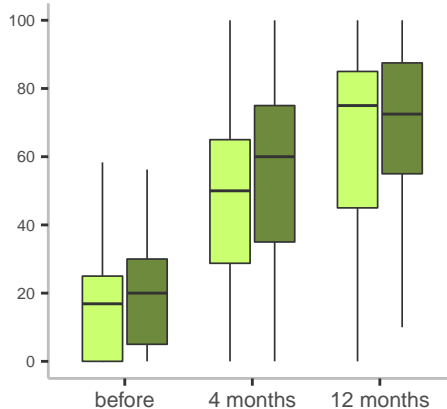

KOOS quality of life

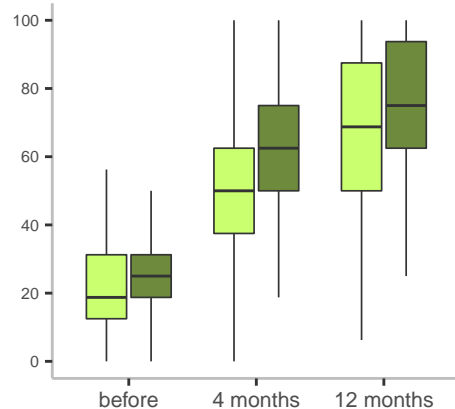

FJS-12

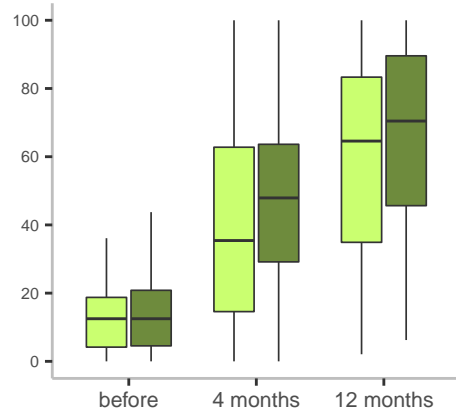

EQ-5D-3L

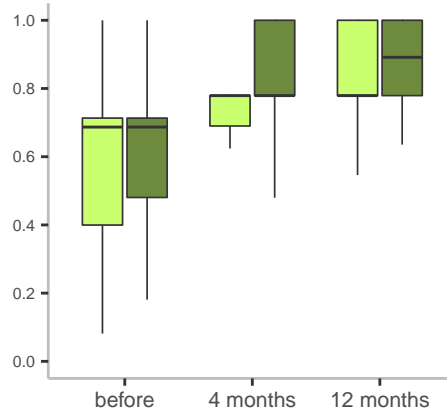

EQ-VAS

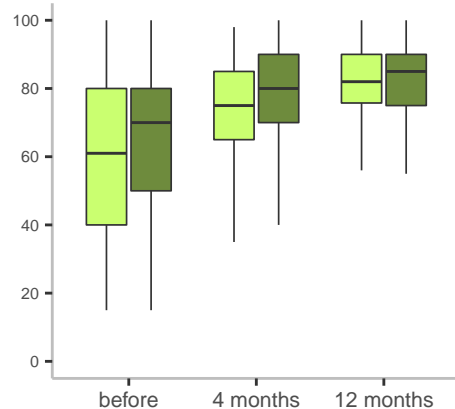

Supplement: Supplementary file 3 — Supplementary file3 Boxplots of KSS and PROMs before and after the surgery: comparison younger and older patients. KSS Knee Society Score, KOOS Knee injury and Osteoarthritis Outcome Score, FJS-12 Forgotten Joint Score, VAS visual analogue scale (PDF 15 KB) [file 167_2022_6900_MOESM3_ESM.pdf]
